# Persistence and spillback of mammal-adapted H5N1 genotype B3.2 viruses among South American seabirds and marine mammals

**DOI:** 10.21203/rs.3.rs-7960151/v1

**Published:** 2025-11-04

**Authors:** Agustina Rimondi, Ralph Vanstreets, Martha Nelson, Valeria Olivera, Luciana Gallo, Alexis Durant, Flavio Quintana, Martin Brogger, Julieta Campana, Simon Dellicour, Thorsten Wolff, Marcela M. Uhart

**Affiliations:** Robert Koch Institute; Karen C. Drayer Wildlife Health Center, One Health Institute, School of Veterinary Medicine, University of California - Davis; National Center for Biotechnology Information, NIH; Instituto de Virología e Innovaciones tecnológicas, Centro de Investigaciones en Ciencias Veterinarias y Agronómicas, INTA-CONICET.; Instituto de Biología de Organismos Marinos (IBIOMAR), Consejo Nacional de Investigaciones Científícas y Técnicas (CONICET).; Karen C. Drayer Wildlife Health Center, One Health Institute, School of Veterinary Medicine, University of California.; Instituto de Biología de Organismos Marinos (IBIOMAR), Consejo Nacional de Investigaciones Científícas y Técnicas (CONICET).; Instituto de Biología de Organismos Marinos (IBIOMAR), Consejo Nacional de Investigaciones Científícas y Técnicas (CONICET).; Wildlife Conservation Society, Argentina Program.; Université; Robert Koch Institute; University of California Davis

## Abstract

The arrival of H5N1 clade 2.3.4.4b genotype B3.2 in South America marks an unprecedented geographic expansion of highly pathogenic avian influenza viruses. The continent remains the only region with sustained H5N1 transmission in wild marine mammals. To investigate the evolution of H5N1 in this novel context, we conducted genomic surveillance in marine wildlife along the northern Patagonian coast in Argentina (August 2023 – February 2024). Phylogenetic analyses revealed two B3.2 subclades: an avian subclade linked to poultry outbreaks in central Argentina, and a marine mammal subclade that persisted locally and repeatedly spilled back into seabirds, causing mortality in terns. Notably, most seabird viruses retained mammalian-adaptive mutations; however, one tern cluster exhibited reversion only at PB2-N701D. These findings suggest the ability of South American seabirds to sustain mammal-adapted H5N1 viruses, potentially enabling long-distance spread and establishment in novel wildlife reservoirs, thereby threatening biodiversity and increasing risks to animal and public health.

## INTRODUCTION

The ongoing panzootic of highly pathogenic avian influenza (HPAI) H5N1 clade 2.3.4.4b represents an unprecedented global challenge. Noted for its high virulence and worldwide prevalence, this clade has caused significant outbreaks in both wild and domestic bird populations, with numerous spillover events into mammals. These outbreaks have resulted in severe ecological disruption and considerable economic losses^1,2^. The global dissemination of this clade has been driven by multiple factors, including migratory bird movements and frequent genetic reassortment events^3-5^, which facilitate viral adaptation, persistence, and evolution across diverse ecological niches.

Of particular concern is the increasing number of documented spillover events into non-human mammalian hosts – including sea lions, fur seals, elephant seals, cats, foxes, skunks, mink, dairy cattle, llama and sheep – across multiple regions^6,7^. In many instances, H5N1 viruses recently detected in mammals often harbor key adaptive mutations in the PB2 polymerase gene (e.g., T271A, Q591K, E627K, M631L, D701N)^8-12^, known to enhance replication efficiency, virulence, and host adaptation^13-17^. These findings underscore the capacity of H5N1 clade 2.3.4.4b viruses to acquire mammalian-adaptive traits following interspecies transmission, reinforcing the need for enhanced genomic surveillance and a deeper understanding of the ecological and molecular mechanisms driving this emergence.

Within clade 2.3.4.4b, the introduction of H5N1 genotype B3.2 viruses (hereafter referred to as H5N1-B3.2) into South America in late 2022 marked a turning point in the global expansion of HPAI H5Nx viruses and represented the first occurrence of these viruses in the Neotropical realm. Following introduction into Peru^18^, H5N1-B3.2 viruses rapidly disseminated through wild birds and marine mammal populations causing large-scale mortality across multiple species in South America^10,19-23^ and later spreading to the Subantarctic and Antarctic regions for the first time^24-26^. This situation has raised serious concerns for wildlife conservation and poultry across the continent.

In early 2023, a distinct subclade of H5N1-B3.2 viruses emerged in Peru or northern Chile, triggering a series of outbreaks in marine mammals, especially pinnipeds (seals, fur seals and sea lions), along the coast of South America^9,10,12,20,23^. We previously demonstrated that viruses from these events formed a unique marine mammal-associated subclade within H5N1-B3.2, harboring key mutations associated with mammalian adaptation (PB2-Q591K and PB2-D701N) that likely facilitated efficient transmission among pinniped populations^9,10^, a finding later confirmed by others^12,20^. This mammal-adapted subclade was also implicated in a severe human case in Chile, emphasizing its zoonotic potential^27^.

During the early spread of these mammal-adapted H5N1-B3.2 viruses in Argentina (August to early October 2023), seabird mortality remained relatively low, with scattered deaths of species such as terns and grebes occurring alongside mass mortality events in pinnipeds^9,10^. Starting in mid-October 2023, however, there was a marked increase in seabird mortality along the northern Patagonian coast. Given the extensive habitat overlap between marine mammals and seabirds in this region, and the importance of monitoring the H5N1-B3.2 viruses as they spread across species and sites, targeted efforts were conducted to detect and characterize circulating strains.

Here, we present a comprehensive genomic and phylogeographic analysis of H5N1-B3.2 viruses recovered between August 2023 and February 2024 from seabirds and marine mammals along the northern Patagonian coast of Argentina. To place these findings in a continental context, we reconstructed the spatial spread of H5N1-B3.2 across South America using a continuous phylogeographic diffusion model. This analysis reveals contrasting dissemination patterns between the avian- and marine mammal-associated H5N1-B3.2 subclades, highlighting notable geographic and temporal differences in their spread. We further demonstrate that the mammal-associated H5N1-B3.2 subclade not only caused mass mortality events in sea lions and elephant seals in Patagonia, but also spilled over into terns and other seabirds. This study provides valuable genomic evidence of the remarkable host plasticity of H5N1-B3.2 viruses, demonstrating its potential to spread across both marine mammal and avian species. Understanding these dynamics is relevant to elucidate the ecology and epidemiology of HPAI H5N1 viruses, particularly when introduced to novel wildlife communities across biogeographical realms.

## RESULTS

### H5N1 outbreaks in marine wildlife in northern Patagonia, Argentina.

**Figure 1:**
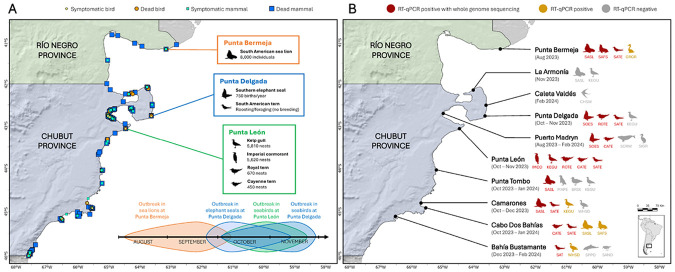
Distribution of suspected cases, key study sites, sampling effort, and detection of H5N1 clade 2.3.4.4b virus in wildlife along the northern Patagonian coast of Argentina. A) Suspected cases of HPAI in birds and marine mammals (symptomatic or deceased) along the coasts of Río Negro and Chubut provinces (legend shown; data compiled by provincial environmental authorities between August 2023 and February 2024) in relation to the three key study sites (Punta Bermeja, Punta Delgada, and Punta León; population sizes derived from the literature^28,30-32^); a conceptual timeline representation of the outbreaks at these key study sites is shown at the bottom. B) Distribution of the sampling effort (period of sample collection is shown in parenthesis) for different wildlife species and diagnostic results (legend is shown). Species are abbreviated as follows: BRSK – brown skua (*Stercorarius antarcticus*), CATE – cayenne tern (*Thalasseus acuflavidus eurygnathus*), CHSW – Chilean swallow (*Tachycineta leucopyga*), GRGR – great grebe (*Podiceps major*), KEGU – kelp gull (*Larus dominicanus*), IMCO – imperial cormorant (*Leucocarbo atriceps*), MAPE – Magellanic penguin (*Spheniscus magellanicus*), ROTE – royal tern (*Thalasseus maximus*), SAND – sanderling (*Calidris alba*), SAFS – South American fur seal (*Arctocephalus australis*), SASL – South American sea lion (*Otaria byronia*), SATE – South American tern (*Sterna hirundinacea*), SIGR – silvery grebe (*Podiceps occipitalis*), SOES – southern elephant seal (*Mirounga leonina*), SORW – southern right whale (*Eubalaena australis*), SPPO – spectacled porpoise (*Phocoena dioptrica*), WHSD – white-headed steamer duck (*Tachyeres leucocephalus*).

Between August 2023 and February 2024, monitoring of wildlife mortality was conducted along the northern Patagonian coast of Argentina (Río Negro and Chubut provinces; Figure 1A). Initial mortality events primarily involved marine mammals, with major outbreaks in South American sea lions (*Otaria byronia*) at Punta Bermeja^9,28^ and southern elephant seals (*Mirounga leonina*) at Punta Delgada and throughout the outer coast of Península Valdés^10,29^. From October 2023 onwards, a pronounced increase in seabird mortality was observed, reaching a maximum in late October at Punta León and mid-November at Punta Delgada (chronological plot in Figure 1A). As previously reported^10^, over 400 birds, mostly South American terns (*Sterna hirundinacea*), were found dead at Punta Delgada from mid-October to November 2023. In parallel, mass mortality of seabirds occurred at Punta León, with over 2,100 birds found dead over a one-month period starting in early October. Cayenne terns (*Thalasseus acuflavidus eurygnathus*) and royal terns (*Thalasseus maximus*) were most affected (>95% of carcasses), with smaller number of kelp gulls (*Larus dominicanus; c.* 50 individuals), imperial cormorants (*Leucocarbo atriceps; c.* 10 individuals), and South American terns (*c.* 10 individuals). Prior to death, affected seabirds exhibited neurological signs and respiratory distress suggestive of HPAI H5Nx infection. At Punta León, five South American sea lions, four southern elephant seal pups, and one juvenile South American fur seal (*Arctocephalus australis*) were also found dead. Although these animals were not sampled and no direct evidence links their deaths to H5N1, their presence at the site of a confirmed H5N1 outbreak in seabirds raises the possibility that they were affected by the same epizootic (see below). Additionally, sporadic mortality of marine birds was recorded, including cases in great grebe (*Podiceps major*) and white-headed steamer duck (*Tachyeres leucocephalus*) in which H5N1 infection was confirmed (Figure 1B; Supplementary File 1).

### H5N1-B3.2 marine mammal subclade detected in seabirds and marine mammals.

Covering ten sites (Figure 1B), we collected oropharyngeal, tracheal, lung, brain, and rectal/cloacal samples from 93 deceased or symptomatic animals. These included 43 mammals from five species and 50 birds from eleven species (Supplementary File 1). Incorporating our previously published data^9,10^, we detected influenza A virus (IAV) in three mammal and seven bird species at eight sites. Sequencing of newly identified IAV-positive samples collected between October 2023 and February 2024 resulted in the submission of sixteen complete and three partial genome sequences to GenBank (accession numbers PV717645-PV717785, and PV717834). All IAV genome sequences obtained from marine wildlife in the northern Patagonian coast (in total 27 complete and 6 partial H5N1 genomes) correspond to the marine mammal-associated H5N1-B3.2 subclade.

### Distinct spatial diffusion patterns of H5N1-B3.2 in avian and marine mammal hosts in South America.

**Figure 2:**
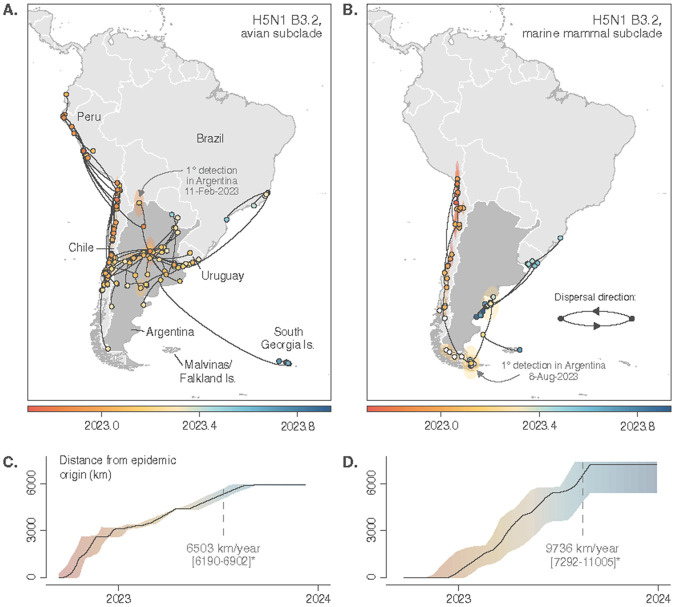
Spatial dissemination of H5N1-B3.2 virus in South America in birds and marine mammals. Curves and colored dots represent phylogenetic branches (lineage dispersal events) and nodes of the maximum clade credibility (MCC) tree inferred by a continuous phylogeographic analysis. Inferred tree node locations and the associated 80% highest posterior density (HPD) polygons – refiecting the uncertainty associated with the Bayesian phylogeographic inference – are colored according to their time of occurrence, progressing from red (2022) to blue (2024) for the B3.2 avian subclade (A) and B3.2 marine mammal subclade (B). White dots indicate the position of tip and internal nodes associated with unsampled taxa included in the continuous phylogeographic analysis and corresponding to marine mammal outbreaks in southern Patagonia for which genomic sequences were not available (see Methods). Below, plots quantify expansion dynamics for the (C) avian subclade and (D) marine mammal subclade by showing the progression of the maximal distance of the epidemic wavefront from its geographic origin (the MRCA) over time. Solid lines show median distances and shaded areas represent the 95% HPD intervals. (*) The annotated values are the median wavefront velocities in kilometers per year during the initial invasion phase.

A continuous phylogeographic diffusion model in two-dimensional space (latitude and longitude) was used to reconstruct the geographic dispersal of H5N1-B3.2 viruses in South America. This analysis enables the detailed mapping of geographic spread for two distinct host-associated H5N1-B3.2 subclades circulating concurrently in the region, the avian and the marine mammal subclades (Figures 2A and 2B respectively; Supplementary Figure 1).

The H5N1-B3.2 avian subclade spread rapidly from Peru/Chile across the South American mainland, overpassing the Andes Mountains west-to-east before being detected in a wild goose in northwest Argentina in February 2023. From there, the virus spread quickly throughout northern and central Argentina, frequently spilling over into domestic birds and causing sustained outbreaks on poultry farms and backyard flocks between February and June 2023. The avian subclade expanded from Argentina northeastward into Uruguay and Brazil, eastward over 2,000 km from inland Argentina to South Georgia (Islas Georgias del Sur), and bidirectionally between Argentina and Chile to the west (Figure 2A; MOV_1). In contrast, the H5N1-B3.2 marine mammal subclade followed an extended coastal dissemination route, initially moving north-to-south along the Pacific coast of Peru, then continuing southward over ~6,000 km of Chilean coastline before reaching the southern tip of South America. From there, it turned north along Argentina’s Atlantic coast, reaching Uruguay, Brazil, and also spreading eastwards to the Falkland Islands (Islas Malvinas) in the Atlantic Ocean (Figure 2B; MOV_2).

The H5N1-B3.2 marine mammal subclade exhibited a relatively lower weighted diffusion coefficient (1,310 km^2^/day, 95% HPD = [864, 1958]) compared to the avian subclade (1,764 km^2^/day, 95% HPD = [1522, 2040]), although their highest posterior density (HPD) intervals overlapped. Despite this, the marine mammal subclade tended to progress relatively faster from its epidemic origin than the avian subclade, as reflected by higher wavefront velocities relative to the avian subclade (Figure 2C-D). However, the wavefront comparison should be interpreted cautiously, as the maximal distance from the epidemic origin was estimated differently for each subclade due to the non-linear direction of the marine mammal subclade (see Methods), and because wild bird sampling across South America is uneven. Additionally, we find that the velocity of the marine mammal wavefront is similar on both the Pacific coast and Atlantic coast (Figure 2D).

### Evolution and spread of the H5N1-B3.2 marine mammal subclade in Argentina.

**Figure 3:**
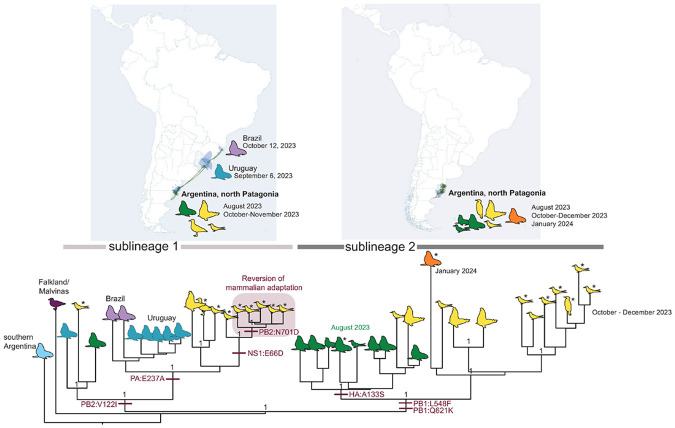
H5N1-B3.2 marine mammal subclade in Argentina. Maximum clade credibility (MCC) tree inferred for 42 fully sequenced H5N1-B3.2 viruses collected in birds and marine mammals in Argentina, Brazil, Uruguay, and Falkland Islands (Islas Malvinas) between August 2023 and January 2024. Cartoons represent the host species (see Figure 1, tree with tip labels is available in Figure 4). Cartoons are shaded by location (teal = Uruguay, light purple = Brazil, dark purple = Falkland Islands (Islas Malvinas); light blue = southern Argentina). Asterisks indicate samples collected for this study. Sampling dates in northern Patagonia, Argentina, are further differentiated by color (green = August 2023; yellow = October to December 2023; orange = January 2024). Sublineages 1 and 2 are labeled on the tree, and maps for each are provided above. Green lines indicate the routes of spatial dissemination of H5N1-B3.2 viruses inferred from the MCC tree, representing coastal outbreaks in marine mammals and seabirds.

**Figure 4:**
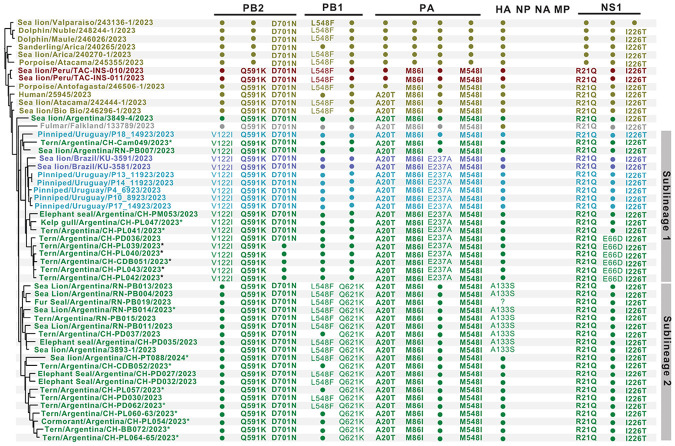
Signature mutations defining the H5N1-B3.2 marine mammal subclade viruses. Amino acid changes are listed for mutations that emerged in the marine mammal subclade of the HPAI H5N1 (2.3.4.4b, genotype B3.2) viruses since late 2022 (Figure 2B). Virus names and associated mutations are colored by country. A question mark indicates that no sequence data is available for that virus at that position. HA mutations are referenced according to H5 numbering. Asterisks indicate samples collected for this study (black asterisks denote viruses with the N701D reversion in PB2).

The MCC tree indicates that the H5N1-B3.2 marine mammal subclade diverged into two sublineages that co-circulated over time and space in Argentina (Figure 3). Both sublineages share a conserved core set of seven mutations (PB2-Q591K, PB2-D701N, PA-A20T, PA-M86I, PA-M548I, NS1-R21Q, and NS1-I226T), previously associated with this subclade^9,10,12^ (Figure 4). Sublineage 1 is characterized by the PB2-V122I substitution, often accompanied by PA-E237A, whereas sublineage 2 is defined by the PB1-Q621K mutation. Additionally, most sublineage 2 viruses harbor the PB1-L548F substitution, although partial reversion to the ancestral 548L residue was observed in 7 of 20 (35%) viruses within this group. The HA gene exhibited limited variation, with only a single HA mutation (A133S) identified in eight viruses from sublineage 2 associated with the initial mass mortality events at Punta Bermeja and Punta Delgada (Figures 1A, 3-4). This contrasts with the stability of core mutations in the polymerase and NS1 genes identified in H5N1-B3.2 marine mammal viruses.

Spatially, both sublineages were detected in Argentina during the outbreaks that occurred in August 2023 and October-November 2023 in marine mammals and seabirds (Figure 3; Supplementary File 1 and Figure 2). Sublineage 1 spread to Uruguay (September 2023) and Brazil (October 2023), whereas Sublineage 2 was detected only in Argentina, with the last detection in February 2024 (Supplementary Figure 3). Phylogenetic analysis of 19 seabird-derived H5N1 viruses (15 newly generated and 4 previously reported^9,10^) showed that nine viruses belonged to sublineage 1 and ten to sublineage 2, confirming that both sublineages infect multiple seabird species. Importantly, none of these seabird viruses clustered with the avian subclade responsible for poultry and inland wild bird outbreaks earlier that year in Argentina (Figure 2A). Instead, all seabird-derived viruses clustered within the H5N1-B3.2 marine mammal subclade (Figure 2B) and grouped closely with sequences from infected sea lions, fur seals, and elephant seals, supporting multiple independent spillback events from marine mammals to seabirds in northern Argentine Patagonia (Figures 3-4).

### Reversion of the PB2-D701N mammalian-adaptive mutation in a subset of H5N1-B3.2 viruses from terns.

**Figure 5:**
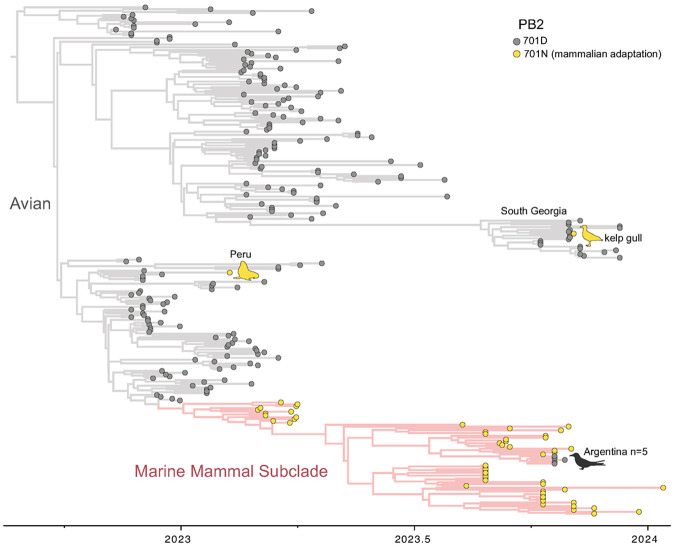
H5N1 HPAI (2.3.4.4b, genotype B3.2) viruses in South American marine mammals and birds. Time-scale MCC tree inferred for the concatenated genome sequences (~13kb) of 267 H5N1 viruses belonging to genotype B3.2 collected in five South American countries (Argentina, Brazil, Chile, Peru, and Uruguay) and in South Georgia and the Falkland Islands (Islas Malvinas). Branches in the marine mammal subclade are shaded pink. Tips are shaded by the amino acid position at site 701 in the PB2 segment: grey = D (Asp) and yellow = N (Asn). Labels and cartoons are provided for mutations outside the marine mammal subclade and reversions and the 701D reversion in Argentina terns. XML file and tree file available in GitHub: https://github.com/mostmarmot/Argentina_HPAI_Oct23-Feb24.

Among the newly generated H5N1-B3.2 seabird-derived sequences in this study, five tern viruses within sublineage 1 cluster together on the phylogenetic tree and share a reversion of the mammalian-adaptive PB2-D701N mutation to the ancestral 701D residue (Figures 3-5). Four viruses (cayenne tern, n=2; royal tern, n=2) with the N701D reversion were collected on 19 October 2023 in Punta León and one virus (cayenne tern) was collected on 27 October 2023 in Cabo Dos Bahías (about 230 km to the south; Supplementary Figure 2). The statistical support for this cluster of viruses is high (posterior probability = 1.0, Supplementary Figure 4). As a sensitivity test, the five tern viruses with the N701D reversion continued to cluster together even when position 701 in PB2 was masked (all trees provided in GitHub, https://github.com/mostmarmot/Argentina_HPAI_Oct23-Feb24). In addition, the five viruses in the N701D reversion cluster share a second unique mutation: NS1-E66D (Figure 4), further supporting that there was bird-to-bird transmission after an initial mammal-to-bird spillback event. Variant detection analyses indicate that approximately 60-93% of sequence reads in these samples have the N701D reversion, indicating the presence of mixed viral populations within these samples (Supplementary Figure 4). Another tern virus (from a South American tern in Camarones) was positioned outside the reversion cluster but also had low percentages of reads with the N701D reversion (22%). Other clusters of avian viruses in the marine mammal subclade lacked avian mutational signatures or other strong evidence of bird-to-bird transmission (Figures 4-5).

## DISCUSSION

The incursion of HPAI H5N1-B3.2 into South America has caused multiple outbreaks in domestic poultry and unprecedented mortality across diverse wild bird and marine mammal species, raising urgent conservation concerns and severe ecological consequences, many of which remain challenging to quantify^28,33-35^. Notably, South America is currently the only continent where HPAI has demonstrated sustained transmission in wild marine mammals^10,12^. Our study shows that the distinct H5N1-B3.2 marine mammal subclade – responsible for widespread mortality in pinnipeds across five countries in 2023, including Argentina^9,10,12,20,23^ – persisted in northern Patagonia until at least February 2024. This subclade has also repeatedly spilled back into seabirds. Moreover, one phylogenetic transmission cluster of five tern viruses shares the PB2-N701D reversion back to the avian-preferred aspartic acid (D, Asp). The five tern viruses also share a distinctive NS1 mutation (E66D), supporting avian transmission of H5N1-B3.2 viruses bearing mammalian adaptations, most notably PB2-Q591K, which has not reverted back to an avian form. Whether wild birds in South America will continue to sustain H5N1-B3.2 viruses carrying mammalian adaptations, or whether these mutations impose fitness costs in avian hosts, remains an open and important question.

By tracking the ongoing evolution of the H5N1-B3.2 marine mammal subclade in Argentina from August 2023 to February 2024, we identified two distinct sublineages with unique genetic markers and different geographic distributions. Notably, both sublineages infect a broad range of hosts, including marine mammals and seabird species (such as terns, cormorants, and gulls). Moreover, these sublineages share core mutations in PB2 (Q591K, D701N), PA (A20T, M86I, M548I), and NS1 (R21Q, I226T), which have been consistently maintained in H5N1-B3.2 marine mammal subclade viruses, likely enhancing mammal-to-mammal transmission^10,12^. Most of these key mutations occur in the polymerase genes – particularly PB2 – and reflect critical adaptive changes that enhance viral replication efficiency and promote host adaptation in marine mammals, as previously observed in other mammalian H5N1 infections^8,11,36^, underscoring the central role of PB2 evolution during the early stages of avian-to-mammal adaptation.

It remains unclear whether the adaptive advantages of H5N1-B3.2 marine mammal viruses arise primarily from the PB2-Q591K and PB2-D701N mutations alone or from their combined effect with the PA and NS1 mutations. Experimentally, a mouse-adapted HPAI H5N8 virus rapidly acquired the PA-M86I mutation alongside mammalian-adaptive PB2 markers^37^, leading to enhanced polymerase activity compared to the wild-type virus. Moreover, NS1 mutations may also contribute to host adaptation by modulating innate immune responses^38,39^. The NS1-R21Q mutation impairs NS1 antagonism of RIG-I signaling, thereby enhancing innate immune activation^40^. Although the function of the NS1-I226T mutation is less clear, its location within the fiexible C-terminal domain of NS1 – known for interacting with host factors and undergoing post-translational modifications^41^ – suggests a potential role in immune evasion or viral replication. Therefore, given the zoonotic potential^27^ and transmissibility in ferret models^42^ of H5N1-B3.2 marine mammal viruses, additional *in vitro* and *in vivo* studies are needed to clarify the role of NS1 mutations, together with polymerase mutations, in viral replication, virulence, and mammalian transmission. Remarkably, the H5N1-B3.2 marine mammal subclade viruses exhibit minimal genetic variation in the HA gene. This contrasts with previous observations of H10N7 viruses isolated from seals in Europe, which accumulated HA mutations associated with enhanced transmissibility and mammalian adaptation^43,44^. The limited HA variability in the H5N1-B3.2 marine mammal subclade likely reflects evolutionary constraints aimed at preserving a receptor-binding domain compatible with infection of diverse wild marine mammal and bird species.

**Figure 6:**
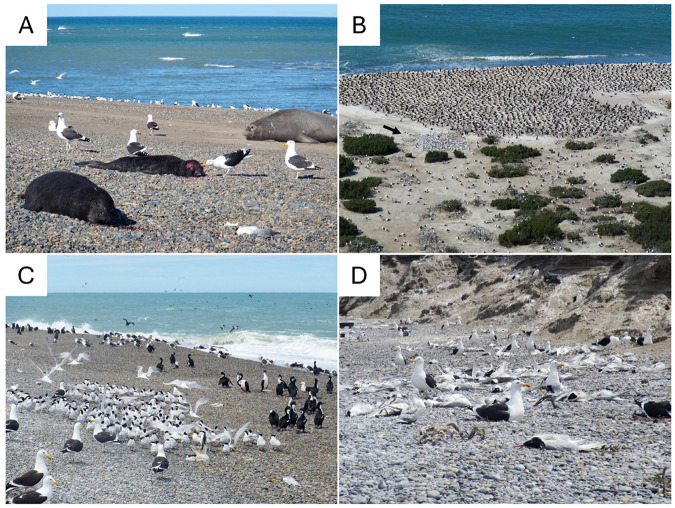
Close proximity of marine wildlife at Punta León. A) Kelp gulls scavenging on two carcasses of southern elephant seal pups that died on the beach in front of the seabird colony. B) Overview of the seabird colony, with the sub-colonies of imperial cormorants (top), royal and cayenne terns (arrow), and kelp gulls (bottom). C) Aggregations of kelp gulls, royal and cayenne terns and imperial cormorants on the beach in front of the seabird colony. D) Kelp gulls nesting on the beach among hundreds of tern carcasses. Photo credits: Ralph E. T. Vanstreels.

A notable finding of this study is that mortality events in seabirds from northern Patagonia were caused by viruses belonging to the H5N1-B3.2 marine mammal subclade rather than the avian subclade. Ecologically, these subclades exhibit little overlap in geographic distribution and host range within Argentina. Although occasional spillover of H5N1-B3.2 marine mammal viruses into seabirds has been sporadically reported^9,10,12,20^, the high frequency of such spillover events reported here is distinctive. In Argentina, genetic sequencing confirmed infections by the mammalian subclade in twenty birds across at least five seabird species – imperial cormorant, kelp gull, cayenne tern, royal tern, and South American tern. Additionally, two more species – great grebe and white-headed steamer duck – showed evidence of infection, although complete genomes could not be obtained. The elevated detection rate likely results from a combination of focused efforts to primarily sample deceased animals, and the ecological interactions between marine mammals and seabirds in this region. While precise transmission pathways in South American marine wildlife remain unresolved, interspecies interactions – such as gull scavenging of pinniped carcasses and nesting on beaches amidst hundreds of tern carcasses – likely facilitate marine mammal-to-bird spillover events (Figure 6A and D). These events may occur via direct contact or indirectly through environmental contamination, underscoring the complexity of cross-species viral transmission in marine ecosystems. Moreover, the presence of mixed-species seabird colonies and the shared use of coastal and marine habitats likely provide ample opportunities for subsequent interspecific bird-to-bird transmission following initial spillovers (Figures 6B-D). Quantifying the frequency and ecological consequences of these transmissions is critical for understanding the evolutionary dynamics and epidemiological risks posed by H5N1 in multi-host systems.

Interestingly, our analyses reveal that reversions in core mutations were remarkably uncommon in H5N1-B3.2 marine mammal subclade viruses isolated from avian hosts: only a small subset of avian-derived viruses from sublineage 1 exhibit reversion at PB2-701, while avian-derived viruses from sublineage 2 show no reversions. This suggests that the mammalian-adaptive mutations of this subclade are well adapted to support infection in both avian and mammalian species – and transmission in mammals, and potentially in birds. This contrasts with recent experimental work demonstrating an asymmetric spillover dynamic, in which H5N1 clade 2.3.4.4b viruses adapted to gulls infected minks subclinically and rapidly acquired mammalian-adaptive mutations (e.g., PB2-T271A), whereas spillback from minks to gulls was not observed^36^. Such experimental findings support the hypothesis that selective pressures for HPAI infection in birds and mammals can be zero-sum or asymmetric, with mammalian adaptations potentially compromising fitness in avian hosts^45,46^. However, our data suggest that the H5N1-B3.2 marine mammal subclade viruses may represent an exception – or at least a more complex scenario – in which mammalian-adaptive mutations can persist without obvious fitness costs in seabird hosts. This persistence may be influenced by polygenic factors shaping adaptation to different hosts, together with a natural setting that provides abundant opportunities for mammal-bird transmission. This observation raises important questions about the evolutionary plasticity of these viruses – specifically, their capacity to adapt to and circulate within diverse host species – and emphasizes the urgent need for continued surveillance given their potential for sustained transmission among birds and at bird-mammal interfaces.

In conclusion, our work underscores the intricate interplay between host adaptation and viral evolution, highlighting the critical importance of genomic surveillance in wildlife populations to accurately characterize H5N1 viruses and detect emergent variants in a timely manner. Our findings reveal that although the H5N1-B3.2 marine mammal subclade viruses show a series of mutations that enhance their ability to infect and transmit among pinnipeds, this does not necessarily limit their ability to infect birds. Instead, the viruses of this subclade may function as a broader “marine wildlife subclade,” capable of infecting multiple host species without strict host specificity. This host niche expansion towards seabirds observed since late 2023 contrasts with earlier outbreaks where these viruses were almost exclusive to marine mammals, revealing knowledge gaps on the evolutionary and ecological drivers that shape host range dynamics and spillover events. Although the H5N1-B3.2 marine mammal subclade viruses appear to have dissipated from South America in early 2024 (the last detection was on 12 February 2024, A/cayenne tern/Argentina/CH-PM093/2024 sequenced as part of this study; Supplementary Figure 3), they successfully spread to the Falkland Islands (Islas Malvinas)^25^ and to the Antarctic Peninsula^47^ before vanishing from South America. Given their remarkable host plasticity, coupled with the high mobility of wild birds and marine mammals, the risk of further dissemination through wildlife to other continents (e.g. Indian Ocean and Oceania) and epidemiological compartments (e.g., poultry, terrestrial animals and humans) cannot be overlooked.

The rapid, transnational spread of H5N1-B3.2 subclade viruses among both marine mammals and birds throughout South America, as well as the Subantarctic and Antarctic regions, reinforces the urgent need for a coordinated international One Health framework for genomic surveillance. To fully explain the mechanisms underlying viral host adaptation, experimental studies – including *in vitro* assays of viral replication and immune evasion, as well as *in vivo* models assessing pathogenesis and transmission dynamics – are required. This integrated strategy is essential to unravel the ecology, evolutionary dynamics, and host range of H5N1 viruses, and to inform risk mitigation strategies aimed at protecting wildlife, domestic animals, and human health.

## MATERIAL AND METHODS

### Sample collection.

A team of trained veterinarians wearing full personal protective equipment collected samples from affected animals. Swabs (oropharyngeal, cloacal/rectal, tracheal, lung and brain) were collected from South American fur seal, South American sea lion, southern elephant seal, southern right whale (*Eubalaena australis*), spectacled porpoise (*Phocoena dioptrica*), brown skua (*Stercorarius antarcticus*), cayenne tern, Chilean swallow (*Tachycineta leucopyga*), kelp gull, imperial cormorant, Magellanic penguin (*Spheniscus magellanicus*), royal tern, sanderling (*Calidris alba*), South American tern, silvery grebe, and white-headed steamer duck found dead between August 2023 and February 2024 in Chubut province, Argentina. Carcass preservation was scored using a standardized system^48^ and is detailed in Supplementary File 1. Additionally, fecal swabs were collected from one live southern elephant seal that was apparently healthy, and oropharyngeal and cloacal swabs were collected from live birds (1 brown skua, 2 kelp gulls, 2 Magellanic penguins) presenting with neurological signs (inability to walk or fly, tremors, repetitive head movements).

Swabs were placed in cryotubes containing 1 mL of DNA/RNA Shield (#R1100-250, Zymo Research, Irvine, CA, USA) for inactivation, and stored in a cooler with icepacks, then transferred to −80°C within 24 hours.

### Virus detection.

Viral RNA was extracted from 140 μL of swab suspension using the QIAamp Viral RNA Mini Kit (#52904, Qiagen, Valencia, CA, USA). RNA was eluted in a final volume of 60 μL and stored at −80°C. For cDNA synthesis, 15 μL of viral RNA was used with random hexamers in a final reaction volume of 30 μL, employing the High-Capacity cDNA Archive Kit (#4368813, Applied Biosystems, Foster City, CA, USA). The cDNA was then tested for the presence of IAV using RT-qPCR. Reactions were conducted with TaqMan Universal PCR Master Mix (#4304437, Applied Biosystems) and primers targeting the matrix gene (forward: 5’-GAC CRA TCC TGT CAC CTC TGA-3’, reverse: 5’-AGG GCA TTY TGG ACA AAK CGT CTA-3’, probe: 5’-FAM-TGC AGT CCT CGC TCA CTG GGC ACG-TAMSp-3’)^49^. Amplification was performed on an ABI Prism 7500 SDS (Applied Biosystems). Quantification cycle (Cq) values were used as a proxy for viral RNA load in different samples and to guide the selection of samples for full genome sequencing.

### Full genome sequencing.

The viral genome was amplified from RNA using a multi-segment one-step RT-PCR with the Superscript III High-Fidelity RT-PCR Kit (#12574035, Invitrogen, Carlsbad, CA), following the manufacturer’s instructions. Amplification was performed with the Opti1 primer set (Opti1-F1: 5’-GTT ACG CGC CAG CAA AAG CAG G-3’, Opti1-F2: 5’-GTT ACG CGC CAG CGA AAG CAG G-3’, and Opti1-R1: 5’-GTT ACG CGC CAG TAG AAA CAA GG-3’)^50^. Amplicons were visualized on a 1% agarose gel and purified using Agencourt AMPure XP beads (#A63881, Beckman Coulter, Brea, CA). The concentration of purified amplicons was quantified using the Qubit High Sensitivity dsDNA Kit (#Q32850, Invitrogen) and a Qubit Fluorometer (Invitrogen).

The sequencing library was prepared using the Rapid Barcode Library Kit (#SQH-RBK110.96, Oxford Nanopore, Oxford, UK) and loaded onto the Mk1c sequencer following ONT’s instructions for R.9 flow cells. Real-time basecalling was performed with MinIT (Oxford Nanopore), and automatic real-time division of passed and failed reads was used as a quality control measure, excluding reads with a quality score below 7. Quality-checked reads were then demultiplexed, trimmed for adapters and primers, and mapped. The final consensus genome strains were generated using CLC Genomics Workbench v24.0.1 (Qiagen).

### Phylogenetic analysis.

Sequence alignments were generated for each of the eight segments of the virus genome (PB2, PB1, PA, HA, NP, NA, MP, and NS) using MAFFT v7.49. Previous studies established that a discrete marine mammal subclade of H5N1 clade 2.3.4.4b (genotype B3.2) viruses circulates in South America, descended from the original avian B3.2 subclade^10,12^. To compare the spatial-temporal dissemination B3.2 genotype viruses in South America in the avian versus marine mammal subclades (Figure 2), we performed a time-scaled Bayesian analysis using the Markov chain Monte Carlo (MCMC) method as implemented in the software package BEAST^51^, using GPUs available from the NIH Biowulf Linux cluster (http://biowulf.nih.gov/). The continuous phylogeographic analysis was performed separately on the avian subclade (n = 196 sequences) and marine mammal subclade (n = 49 sequences) and the R package “seraphim” was used for visualization^52,53^. The dataset included genomes from five South American countries (Argentina, Brazil, Chile, Peru, Uruguay) as well as South Georgia and the Falkland Islands (Islas Malvinas). Only sequences for which geographic coordinates (latitude and longitude) were available or could be retrieved were included in the phylogeographic analyses. The avian subclade dataset included isolated spillovers into mammalian hosts, and the marine mammal subclade included spillbacks into avian hosts, such as terns and other seabirds sampled in Argentina for this study. Since phylogeographic inferences are sensitive to sampling bias, we achieved a more realistic spatial reconstruction in marine mammals (avoiding erroneous dispersal across the South American landmass) by including 14 unsampled genomes in the Bayesian inference. These were placed in the southernmost region of Patagonia and were associated with epidemiologically informed sampling times and locations, though no sequence data were available^54^.

For those continuous phylogeographic analyses^55^, we used a relaxed random walk (RRW) diffusion model with a Cauchy distribution to model dispersal velocity heterogeneity, an exponential growth demographic model, a GTR + G model of nucleotide substitution, and an uncorrelated lognormal relaxed molecular clock model. Due to the size of the dataset, we used the high-performance computational capabilities of the NIH Biowulf Linux cluster. The MCMC chain was run separately 3-5 times for each dataset using the BEAGLE 3 library to improve computational performance, until all parameters reached convergence, as assessed visually using Tracer version 1.7.2. At least 10% of the chain was removed as burn-in and runs for the same dataset were combined using LogCombiner v1.10.5. MCC trees were summarized using TreeAnnotator v1.10.5. All MCC trees are available at GitHub: https://github.com/mostmarmot/Argentina_HPAI_Oct23-Feb24, along with a GISAID acknowledgement table for sequences published by other research groups. Visualization of the spatial dynamics and estimation of wavefront velocities were generated using the R package “seraphim”^52^ (Figure 2). The wavefront velocities were estimated differently for the avian and mammalian subclades to account for the distinct non-linearity of the marine mammal wavefront. The wavefront velocity estimate for the avian subclade was directly based on the geographic distance between each node and the most ancestral node of the tree. The wavefront distance and velocity estimates for the marine mammal subclade were based on the assumption that all lineages evolving on the eastern coast did transit by the southernmost location associated with a tree node.

To examine the spatial dynamics of sublineages 1 and 2 within the marine mammal subclade in finer detail (Figure 3), subtrees of the MCC tree inferred for the marine mammal subclade were visualized using SPREAD4 (https://spreadviz.org)^56^. HA mutations were converted to H5 numbering using BV-BRC’s HA numbering tool^57^. An additional MCC tree without spatial information was inferred for all 267 B3.2 genome sequences from South America and surrounding islands to study patterns of the PB2-D701N mutation and reversions (Figure 5). This tree also included the virus A/cayenne tern/Argentina/CH-PM093/2024 that was collected on 12 February, 2024, the latest sequenced virus in our study, for which only a partial genome sequence could be obtained (Supplementary Figure 4).

### Variant detection using CLC Genomics Workbench.

Nanopore and Illumina reads were processed using CLC Genomics Workbench v24.0.1 (Qiagen). Raw data were first quality-checked and trimmed, including adapter and homopolymer trimming, as well as removal of low-quality and short reads (minimum length of 15 base pairs). Quality control (QC) was optimized with a quality threshold of 0.05 and a maximum ambiguity of 2. Structural variants were called under a diploid ploidy assumption using the CLC Structural Variant Caller, with a minimum of two supporting reads per breakpoint and a breakpoint probability threshold of 0.01. To improve the accuracy of variant calls, a local realignment strategy was applied, realigning unaligned ends in multiple passes.

For fixed ploidy variant detection, filters were applied based on a minimum read length of 20 base pairs, with specific quality criteria such as a minimum coverage of 10x, and a minimum frequency of 1%. Final variant annotation was performed using gene and CDS data from our first detection (A/South American sea lion/Argentina/RN-PB004/2023) in our area of study in August 2023, considering amino acid changes and filtering out synonymous variants.

The workflow considered all frequency variants, including low-frequency variants for further analysis. This variation provides valuable insights into the mixing viral population within the samples, even though consensus genome data was obtained. The analysis results were further filtered using parameters to ensure that only high-quality, biologically relevant variants were included, and extracted as track lists and reports for visualization and interpretation.

## Supplementary Material

This is a list of supplementary files associated with this preprint. Click to download.

• SuppFig2sitesandsublineagesREVISED.pdf
• SuppFig1SD14102025.pdf
• SuppFile1.xlsx
• ACspreadgl.mov
• SuppFigureLegends.docx
• SuppFig3lastmmamal.subcladedetection.pdf
• MMspreadgl.mov
• SuppFig4percentagePB2701Dreversion.pdf

## Data Availability

The sequence data generated in this study have been deposited in GenBank under accession codes PV717645-PV717785, and PV717834.
